# Ensemble averaging for categorical variables: Validation study of imputing lost data in 24-h recorded postures of inpatients

**DOI:** 10.3389/fphys.2023.1094946

**Published:** 2023-01-26

**Authors:** Takayuki Ogasawara, Masahiko Mukaino, Hirotaka Matsuura, Yasushi Aoshima, Takuya Suzuki, Hiroyoshi Togo, Hiroshi Nakashima, Eiichi Saitoh, Masumi Yamaguchi, Yohei Otaka, Shingo Tsukada

**Affiliations:** ^1^ NTT Basic Research Laboratories and Bio-Medical Informatics Research Center, NTT Corporation, Atsugi, Japan; ^2^ Department of Rehabilitation Medicine I, School of Medicine, Fujita Health University, Toyoake, Japan; ^3^ Department of Rehabilitation Medicine, Hokkaido University Hospital, Sapporo, Japan; ^4^ Department of Rehabilitation, Fujita Health University Hospital, Toyoake, Japan; ^5^ NTT Device Innovation Center, NTT Corporation, Atsugi, Japan

**Keywords:** acceleration, missing data imputation, rehabilitation monitoring, stroke, wearable sensors and equipment

## Abstract

Acceleration sensors are widely used in consumer wearable devices and smartphones. Postures estimated from recorded accelerations are commonly used as features indicating the activities of patients in medical studies. However, recording for over 24 h is more likely to result in data losses than recording for a few hours, especially when consumer-grade wearable devices are used. Here, to impute postures over a period of 24 h, we propose an imputation method that uses ensemble averaging. This method outputs a time series of postures over 24 h with less lost data by calculating the ratios of postures taken at the same time of day during several measurement-session days. Whereas conventional imputation methods are based on approaches with groups of subjects having multiple variables, the proposed method imputes the lost data variables individually and does not require other variables except posture. We validated the method on 306 measurement data from 99 stroke inpatients in a hospital rehabilitation ward. First, to classify postures from acceleration data measured by a wearable sensor placed on the patient’s trunk, we preliminary estimated possible thresholds for classifying postures as ‘reclining’ and ‘sitting or standing’ by investigating the valleys in the histogram of occurrences of trunk angles during a long-term recording. Next, the imputations of the proposed method were validated. The proposed method significantly reduced the missing data rate from 5.76% to 0.21%, outperforming a conventional method.

## 1 Introduction

Continuous recordings of daily activities for more than 24 h have been used in a variety of studies on healthcare (Willetts et al., 2018), rehabilitation (Barrett et al., 2018; Simpson et al., 2021), preventive medicine (Rosenberger et al., 2016; Hayase, 2020), and telemedicine (Gurchiek et al., 2019). These studies have used inertial sensors such as acceleration sensors and gyroscopes, or heart-rate sensors. In particular, acceleration sensors have been often used in studies on physical activity monitoring (Qi et al., 2018; Bursais et al., 2022; Huber et al., 2022; Werner et al., 2022). They are widely installed in consumer wearable devices and smartphones (Johnston et al., 2019), and features estimated from recorded accelerations such as postures, are useful for understanding the patient’s condition, risks, and lifestyle (Antar et al., 2019; Pohl et al., 2022). However, a recording lasting more than 24 h is more likely to have data losses than a recording lasting only a few hours because of factors such as equipment failure, data-collection limitations, and human error (Cismondi et al., 2013; Lai et al., 2020). As for equipment failures, wearable sensors and smartphones sometimes run out of battery power, and the application software of smartphones is more likely to stop unexpectedly during a long-term recording than during a short one. In particular, many of the consumer-grade wearable devices do not have data storage functions; instead, they send data to a smartphone in real time by using low-power wireless communications. As such, data are sometimes lost when the smartphone is placed in an area the wireless signal cannot reach. Wearable devices are key technologies in the Internet of Things (IoT) and could be a total medical solution offering continuous, objective, and holistic monitoring to alleviate the burden on human caregivers and support decision making (Dian et al., 2020; Stavropoulos et al., 2020). A variety of solutions to the data-loss problem will be required as commoditization of wearable devices proceeds in the medical field.

To solve the problem of data loss, an imputation method using ensemble averaging of heart-rate measurements for more than 24 h has been proposed (Ogasawara et al., 2021). Although the results of that study suggested that the imputation method reduced periods of data loss by up to ten times, the applicable subject of ensemble averaging was limited to quantitative variables such as heart rate. It is not appropriate to take averages of categorical variables such as postures estimated from acceleration data, which are expressed as labels, i.e., ‘lying’, ‘sitting’, ‘standing’, etc. (Karantonis et al., 2006; Yang and Hsu, 2010). Thus, in this study, we decided to develop a new ensemble method to impute lost data of categorical variables by calculating the ratios of days of individual postures taken during the measurement days.

The data imputation methods used in the related work can be classified into statistical or machine learning approaches (Zhang et al., 2019). Multiple imputation is a well-established statistical technique for dealing with missing data in medical research (Rezvan et al., 2015; Murray, 2018). The use of this method over avoiding exclusion of samples with missing data, has been recommended by reviewers of a clinical journal (Ware et al., 2012). Despite the great success of this method, it requires a variety of different features, such as individual demographic characteristics in clinical records. This means that if the research subject is healthy but without any clinical records or if the usable data are limited to only those collected with wearable sensors such as heart rate and acceleration, the effectiveness of multiple imputation is questionable because it may have too few features to work with. On the other hand, machine-learning approaches have been proposed to impute lost data; these methods include decision trees (Gavankar and Sawarkar, 2015), Bayesian models (González-Vidal et al., 2020), artificial neural networks (Okafor and Delaney, 2021), and support vector regression (Li et al., 2018). Machine learning often achieves high accuracy in estimation or prediction problems, but typically hundreds or even thousands of datasets are needed to apply it. It has been pointed out that the opportunities to collect such large datasets are often limited, except in fields of study that use micro arrays or involve gene analyses (Park and Tamura, 2019; Lin and Tsai, 2020). In addition, the complexity of machine-learning models has meant that their interpretability is low. In contrast to this situation, the imputation described in this paper involves imputing lost data by averaging data measured over a period longer than 24 h to give 24 h of imputed time series data. Moreover, whereas the conventional imputation methods are approaches with group subjects with multiple variables, the proposed method is an individual approach with single variable. Here, ensemble averaging is attractive because it does not require additional kinds of features for imputation. It is applicable to small-group experiments to focus on time series data of individuals. In addition, it can be simply interpreted because it is based on averaging.

## 2 Materials and methods

### 2.1 Experimental setup

**FIGURE 1:**
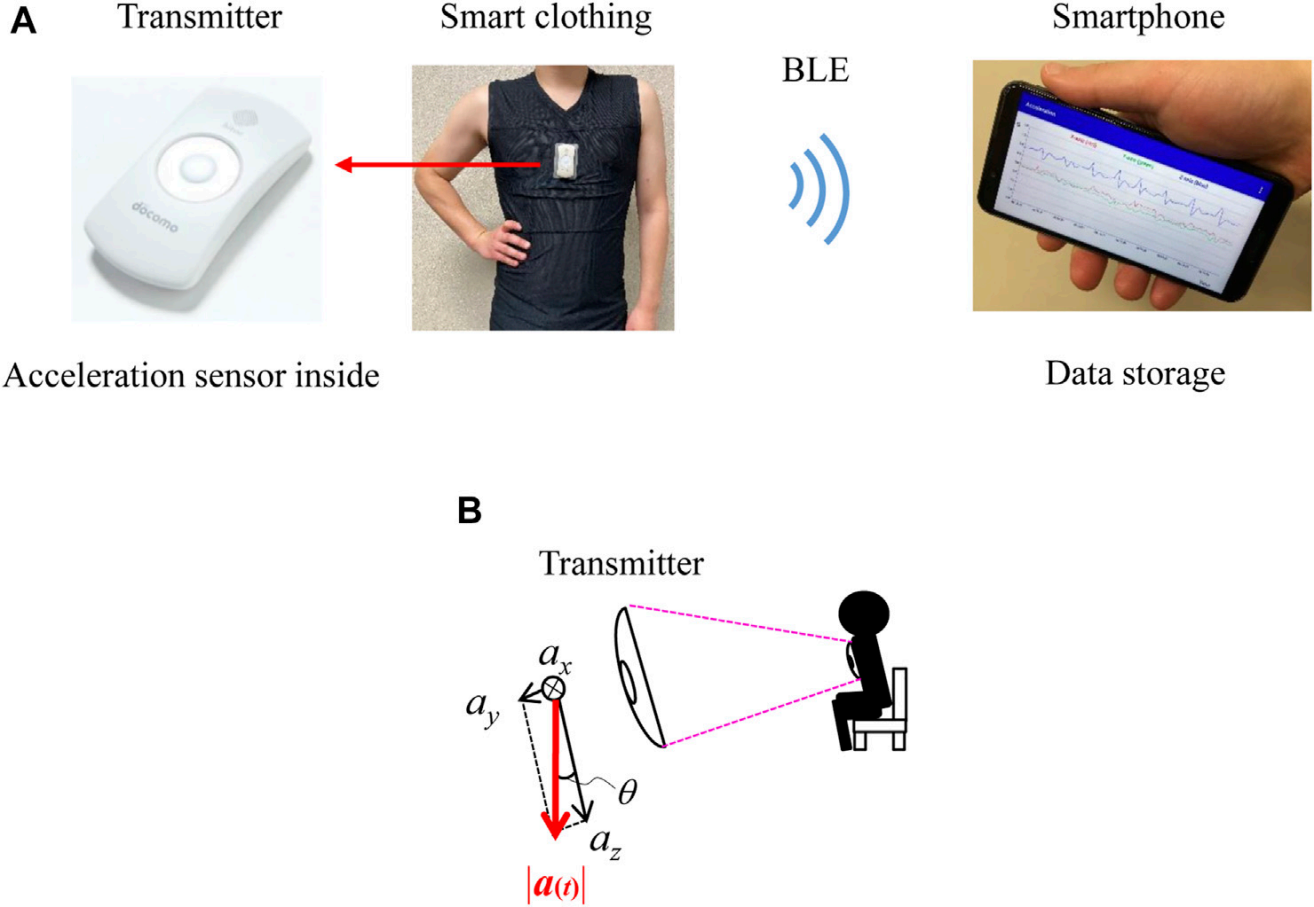
Experimental setup. **(A)** Experimental setup comprised a smartphone and smart clothing with a transmitter. **(B)** Direction of each axis of acceleration.

Figure 1A shows the experimental setup, which comprises a smartphone and smart clothing with a transmitter (Ogasawara et al., 2018; Matsunaga et al., 2019). The ‘hitoe’ transmitter 01 (NTT DOCOMO Inc., Tokyo) is equipped with a three-axis acceleration sensor. Each axis of acceleration is shown in Figure 1B. The sampling rate of the acceleration sensor is 25 Hz. The clothing (Toray Industries, Inc., Tokyo) is skin-tight to hold the transmitter against the body. The transmitter has no memory for storing the acceleration data and uses Bluetooth Low Energy (BLE) to send the data to the smartphone. Thus, each participant carried a smartphone (AQUOS mini SH-M03; Sharp Corp., Sakai), which recorded their data while they were wearing the smart clothing. The application software installed in the smartphone was created by the authors. The smart clothing of the participants was changed every day.

### 2.2 Participants and datasets

99 stroke hemiparesis patients (59 males and 40 females; mean age, 70.1 ± 13 and 71.6 ± 15 years) participated in this study. The patient subjects agreed to wear the measuring devices during the entire 2-day measurement session after checking the size and fit of the wearable device. Some of them participated in several measurement sessions. In these cases, the 2-day measurement was repeated every 2 weeks. The total number of patient measurements was 306. Note that the required length of the session period for imputing the posture data was determined in a preliminary experiment with healthy participants prior to the study; it was found that acceleration data could be sufficiently imputed with a 2-day measurement session (Supplementary Method S1 and Supplementary Result S1). The study protocol was approved by the Medical Ethics Committee of Fujita Health University (HM17-220). All participants provided written informed consent before participating.

Two datasets were prepared for the validation. One contained all of the acceleration data collected in the 306 measurements. Because the time series of postures varies as a stroke patient with hemiparesis recovers, it included multiple measurement data from the same participant with intervals of 2 weeks. The number of repeats of participation depended on participants and it was from zero (no repeat) to 9 times. The other dataset consisted of selected measurements with limited periods of data loss. In particular, it limitedly contained acceleration data of 172 measurements successfully recorded for over 23 h per day during a 2-day measurement session. The former dataset was used to validate the proposed imputation method and the latter was used for statistical investigations and simulations.

### 2.3 Calculation of posture

We classified the postures simply into ‘reclining’, ‘sitting or standing’, and ‘walking’. These simple postures can be estimated from the angle or declination of the acceleration sensor against the direction of the measured acceleration at the trunk in the sagittal plane (Figure 1B) (Rauen et al., 2018). The angle was calculated using the norm of the acceleration vector, as previous studies proposed (Rauen et al., 2018; Fortune et al., 2014; Jeong et al., 2007), according to the following equation (Fisher, 2010):

$$\theta \left(t\right)=\left\{\begin{array}{cc} \frac{180}{\pi }\cos^{-1}\left(a_{z}\left(t\right)/\left|a\left(t\right)\right|\right) & \;if\;a_{y}\left(t\right)\geq 0\\ {-\frac{180}{\pi }\cos}^{-1}\left(a_{z}\left(t\right)/\left|a\left(t\right)\right|\right) & otherwise \end{array}\right.$$ (1)

where $\left|a\left(t\right)\right|=\sqrt{{a_{x}\left(t\right)}^{2}+{a_{y}\left(t\right)}^{2}+{a_{z}\left(t\right)}^{2}}$ . We detected walking periods by using the previously described method (Ogasawara et al., 2016). This method detects walking steps from the norm of the acceleration by using a rules-based algorithm and distinguishes between walking and non-walking situations. In addition to using this status for walking detection, we included it as one of the posture conditions. That is, when a sitting or standing posture is estimated and the algorithm also detects walking at the same time, we consider this situation to be a walking period.

### 2.4 Ensemble averaging procedure

**FIGURE 2:**
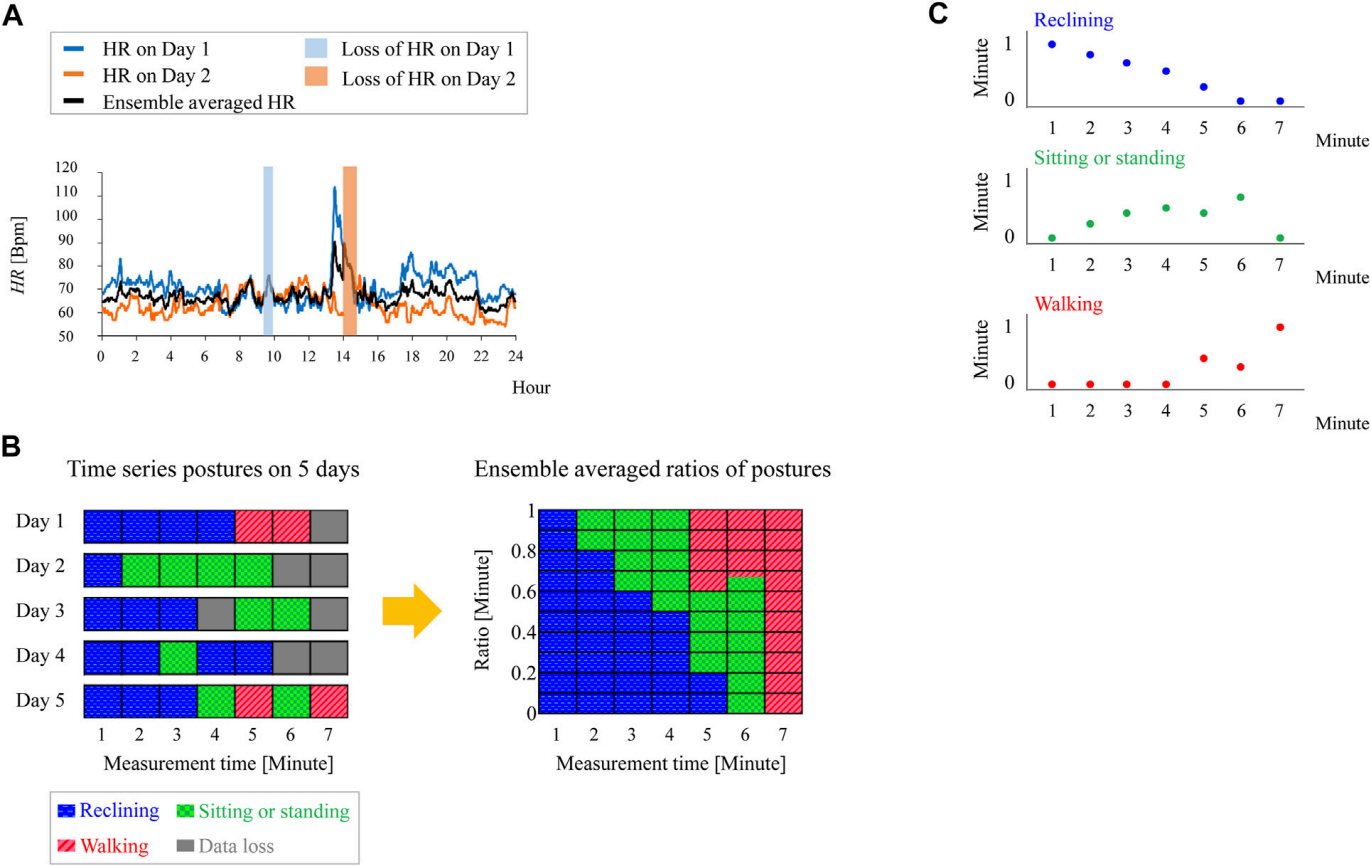
Data imputation using ensemble averaging. **(A)** Imputation of lost heart rate data by ensemble averaging. **(B)** Example of data imputation of posture by using proposed method. **(C)** Ensemble averaged periods of each posture in the case of **(B)**.

Here, we explain the proposed ensemble averaging procedure in Figure 2. First, we briefly summarize the ensemble averaging procedure for quantitative variables that was reported in (Ogasawara et al., 2021). Then, the proposed procedure for categorical variables is introduced.

Let us use heart rate (*HR*) as an example of a quantitative variable. We denote the heart rate on the experimental days during which the measurement was carried out as ${HR}_{EA}\left(t\right)$ , where *d* and *t* represent the day and time of each measurement, respectively. The ensemble averaged heart rate ${HR}_{EA}\left(t\right)$ is

$${HR}_{EA}\left(t\right)=\frac{\overset{N}{\underset{d=1}{\sum }}{HR}_{d}\left(t\right)}{N\left(t\right)}$$ (2)

where N is the total number of measurement days, and $N\left(t\right)$ is the number of days on which the data could be measured at time t. Note that $N\left(t\right)$ is a variable of t and varies depending on the number of days in which data at t has been lost. An example of ensemble averaging of the heart rate during a 2-day measurement is illustrated in Figure 2A. It shows the case of imputing the 24-h heart rate from 48 h of measured heart rates with lost data. The black line shows HREA(t). HREA(t) was calculated even when only HR1(t) or HR2(t) was successfully measured (when $N\left(t\right)=1$ ) and the measured heart rate was substituted as HREA(t) in these cases. In other words, HREA(t) cannot be calculated only if heart rate data are lost at the same o’clock on all measurement days; consequently, the period of data loss is imputed by (2). It was reported that the period of data loss decreased from 5.7% of 24 h to 0.17% in a validation study using 218 measurement data from 63 stroke inpatients, suggesting the possibility of effective data imputation if it is acceptable to record for periods longer than 24 h (Ogasawara et al., 2021).

To impute lost posture data, we perform ensemble averaging on the periods of each posture. The period of occurrence of each posture at *t* is

$$P_{i}\left(t\right)=\frac{N_{i}\left(t\right)}{f\;N\left(t\right)}$$ (3)

Here, $N_{i}\left(t\right)$ is the number of days that a specific posture occurred at *t*, *f* is the sampling rate of the posture, and *i* is the posture state, i.e., ‘reclining’, ‘sitting or standing’ or ‘walking’.

Figure 2B shows an example of data imputation using (3). In this example, the ensemble averaging was carried out on measurements for 5 days and *f* ^−1^ of 1 min. Let us examine two cases: one in which data losses do not occur and one in which a data loss occurs at certain time *t* on the measurement days.

#### 2.4.1 Case without data loss

As shown on the left in Figure 2B, there is no data loss at *t* = 1, 2, 3, or 5 min on any of the measurement days, meaning that *N*(*t*) = 5 in these cases. At *t* = 5, the number of days for each posture, i.e., $N_{i\left(t\right)}$ , is as follows:

$$\left\{\begin{array}{l} Reclining=1\\ Sitting\;or\;standing=2\\ Walking=2 \end{array}\right.$$

Thus, (3) gives the period of each posture occurrence as:

$$\left\{\begin{array}{l} Reclining=0.2\;\left(\min.\right)\\ Sitting\;or\;standing=0.4\;\left(\min.\right)\\ Walking=0.4\;\left(\min.\right) \end{array}\right.$$

#### 2.4.2 Ensemble averaging procedure

When there are missing data, only the successfully measured data at *t* are counted as $N_{i\left(t\right)}$ . For example, the number of days for each posture at *t* = 6 is:

$$\left\{\begin{array}{l} Reclining=0\\ Sitting\;or\;standing=2\\ Walking=1 \end{array}\right.$$

Then, because *N*(*t*) = 3 in this case, the period of each posture occurrence at *t* = 6 is as follows:

$$\left\{\begin{array}{l} Reclining=0\;\left(\min.\right)\\ Sitting\;or\;standing=0.67\;\left(\min.\right)\\ Walking=0.33\;\left(\min.\right) \end{array}\right.$$

The period of each posture is plotted as a time series in Figure 2C. Unless the postures for the same time of day on all measurement days are lost, the ensemble average can be classified into the above two cases. By summing the time series of ensemble-averaged postures, the total period of each posture in 24 h $T_{i}$ is:

$$T_{i}=\int^{t}P_{i}\left(t\right)dt=f^{-1}\int^{t}\frac{N_{i}\left(t\right)}{N\left(t\right)}dt$$ (4)

### 2.5 Statistical data analysis

To compare the proposed and conventional methods of data imputation, statistical analysis were conducted. First, we conducted a Shapiro-walk test to investigate the normality of data distribution. Then, if the normality of at least one of data groups was not confirmed, a Kruskal-Wallis test was conducted. If there was a significant difference with the Kruskal-Wallis tests, a pair comparison was conducted using a Steel-Dwass test.

## 3 Preliminary analysis

Section 3 investigates two factors that had to be determined in order to validate the proposed imputation method. Section 3.1 investigates the measurement periods to decrease the missing data rate with healthy participants. Section 3.2 investigates thresholds on the trunk angles that distinguish between ‘reclining’ and ‘sitting or standing’ from the measured acceleration data of stroke inpatients.

### 3.1 Determination of period of ensemble averaging

In a previous study, the measurement period for inpatients was determined as the time needed to decrease the missing data rate to less than 1% in 24 h by using imputed time-series data; the results suggested that 2 days is a sufficient measurement period to decrease missing heart-rate data to less than 1% in 24 h when ensemble averaging is applied (Ogasawara et al., 2021). The current study investigated the number of measurement session days needed to decrease the missing posture-data rate to less than 1% by using proposed method on acceleration data. It was found that the median of the missing data rate decreased from 6.1% to 0.14% when the proposed imputation method was applied to 2 days of measurement data on healthy participants. Thus, we decided to use posture data of inpatients recorded during a 2-day measurement session to validate the proposed method on hospital inpatients. See the Supplementary Method S1 and Result S1 for details on this preliminary study.

### 3.2 Determination of thresholds for posture classification

Because the ‘reclining’ and ‘sitting or standing’ postures are static, they can be classified in a rules-based manner with thresholds. In addition, the thresholds can be determined from the distribution of the trunk angles measured during about 5 hour recording with healthy participants (Rauen et al., 2018). However, there are issues to be considered with stroke inpatients. First, the posture of an elderly person may be different from that of a younger or healthy subject. Most stroke patients are elderly, and some have hyperkyphosis. Moreover, inpatients spend time on sloped beds during daytime. These differences may affect the posture estimation. To best of our knowledge, there is no statistical information available on postures of stroke patients during 24 h. Therefore, we determined the thresholds of the trunk angles of stroke inpatients obtained during a 2-day measurement. As shown in Supplementary Figure S2, the thresholds were investigated from the distribution of chest angles of a patient during a 2-day measurement. Supplementary Table S1 lists the averages and 95% confidence intervals of the thresholds. The results in the table suggest 35° as the possible threshold between the prone and upright postures and 143° as the one between upright and supine postures for stroke inpatients. See Supplementary Method S2 and Result S2 for details on this preliminary study.

## 4 Results

We validated the data imputations of the proposed ensemble-averaging method. Then, we examined how the ratios of postures depended on missing data. Finally, we visualized the 24-h behavior of patients by using imputed postures.

### 4.1 Validation of missing data imputation

**FIGURE 3:**
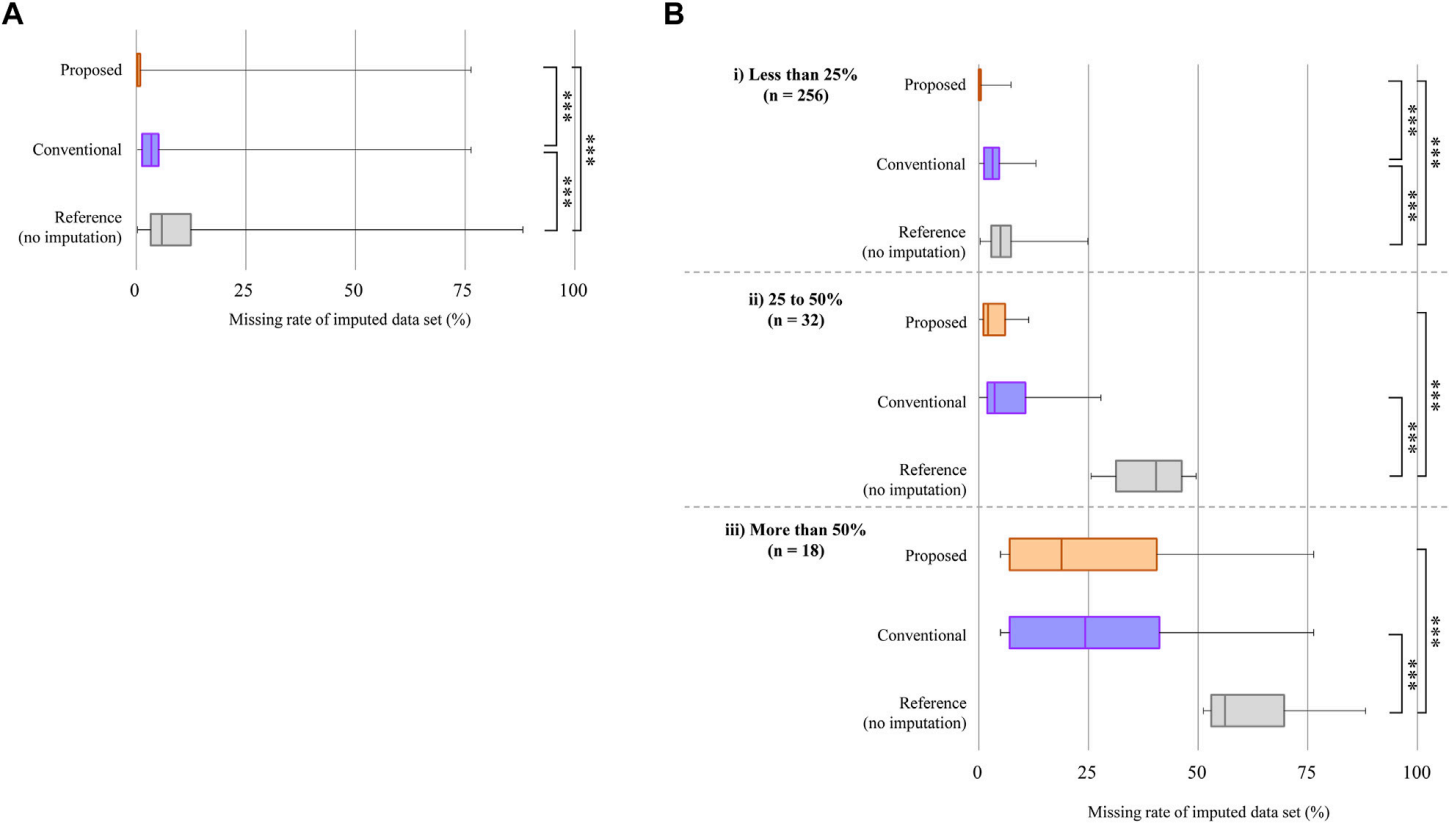
Comparison of imputations of data loss. **(A)** Result with all measurement samples (*n* = 306). The orange box at the top shows the missing data imputed by ensemble averaging (proposed method), the purple box in the middle shows that after selecting the day with the smaller missing data rate between the two measurement days (conventional method), and the gray box at the bottom shows the reference. The edges of the rectangle show the 75th and 25th percentiles. The median is the line inside the box. The median values are shown on each box. Whiskers show the maximum and minimum values. A Kruskal-Wallis test was used to confirm statistically significant differences between the three groups; **p < .001. **(B)** Results of data imputation for different missing data rates without imputation. The horizontal axis shows the missing data rate after applying imputation methods. Kruskal-Wallis tests were used; **p < .001, *p = .008.

Figure 3A shows the results of the imputation of the missing posture data by using the proposed ensemble averaging. Here, all measurement samples (*n* = 306) were used, and the results of the conventional imputation, i.e., selecting data of the day that had less missing data, and those of a reference, namely not performing imputation, are shown. The three methods showed statistically significant differences. The medians of the missing data rate for ensemble averaging and the method of selecting the day with less missing data were 0.21% and 3.47%, respectively. They were smaller than the median of the reference without imputation, 5.76%. A Kruskal-Wallis test was conducted because the normality was not confirmed with any of the groups in Figure 3A and significant differences were found among all three groups (*p* < .001).

To further validate the imputation methods, acceleration measurement samples were classified into three groups according to their missing data rates during a 48-h measurement (Figure 3B). The number of measurement samples is denoted as ‘n’. In all groups, the medians of the missing data rate for the proposed method were smaller than those of the reference with significance shown by a Kruskal-Wallis test. Significant differences were also found between the medians of the conventional method and the reference. In addition, the medians of the missing data rate of the proposed method were smaller than those of the conventional method in all classes. Moreover, significant differences were found in all groups except when the missing data rate was more than 50%.

### 4.2 Validation of ratio of imputed posture

**FIGURE 4:**
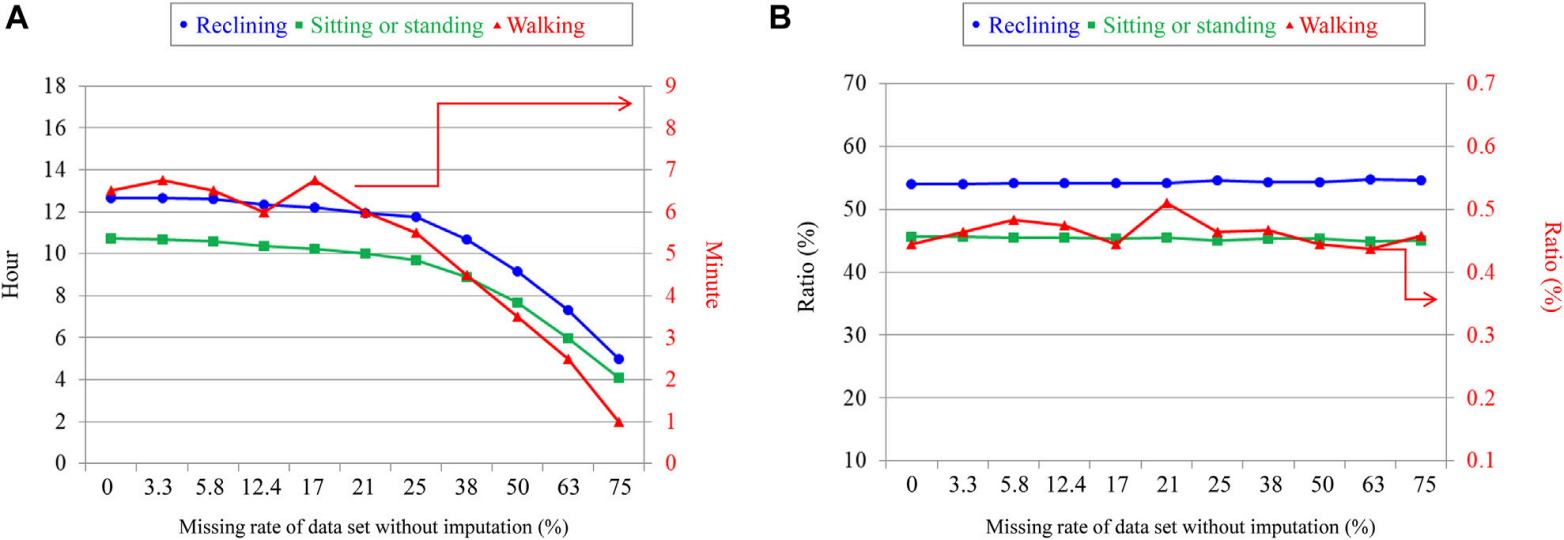
Total periods and ratios of each posture taken during 24 h with imputation. **(A)** Relationship between periods of each posture taken by stroke inpatients and missing data rate. **(B)** Relationship between the periods of each posture over the total activity periods and missing data rate. The vertical axis on the left shows the scale for reclining and sitting and standing postures. The one on the right shows the scale for walking.

To show the effectiveness of the proposed method, we conducted a simulation study by adding artificial data losses to measured data. We chose the dataset mentioned in Section 2, consisting of 172 samples from stroke inpatients successfully recorded for over 23 h per day during a 2-day measurement session. To that dataset, we randomly added artificial losses with a duration of 1 min. Figure 4 shows the results of the simulation. Figure 4A shows the missing data rates of each posture after applying the proposed method; 3.3%, 5.8% and 12.4% on the horizontal axis correspond to the values of the 25th percentile, median, and the 75th percentile of missing data rate of the original dataset during the 2-day measurement session. The values of 3.3%, 5.8% and 12.4% match the edges and middle of the box of the reference in Figure 3A in Section 4.1. Figure 4A suggests that the periods of each posture tended to level off unless the missing data rate was more than 25% during the 2-day measurement session. Otherwise, the periods of each posture got shorter, suggesting that the proposed method was not able to impute data losses with a large missing data rate. Figure 4B shows the ratios of the periods of each posture over the total period of the measured data. The total period was calculated as the sum of the periods of reclining, sitting or standing, and walking. The ratios of the periods of recalling and sitting or standing tended to level off around 54% and 45%, respectively, and they varied within 1% at all missing data rates without imputation. The walking period ratio leveled off around 0.45% and varied within 0.07% at all missing data rates without imputation. These results suggest that the ratios of the three postures tended to be constant and that the proposed ensemble averaging method did not distort the ratios of each posture. In addition to the results shown here, we further examined the effect of the duration of data loss on the proposed imputation method. However, no observable degradation of imputation performance was found when losses with durations longer than 1 min were randomly added to the dataset (Supplementary Figure S4). See Supplementary Method S3 and Result S3 for details.

### 4.3 Visualization of 24-h behavior using imputed posture

**FIGURE 5:**
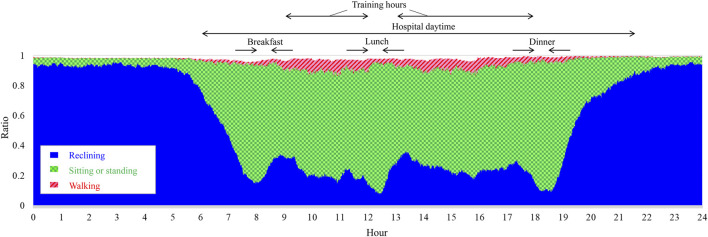
Postures of stroke patients during 24 h. The vertical axis shows the population ratio, where 1 represents the total number of participants, *n* = 306.

Time series of stroke inpatients’ activity during 24 h are shown in Figure 5. The vertical axis is the population ratio, where 1 represents the total number of measurements. The ratio of sitting or standing and walking tended to increase in the daytime and decrease at night. In particular, the ratio of reclining clearly decreases at mealtime but it rapidly increases just after mealtime, suggesting that stroke inpatients who have hemiparitic bodies tend to rest often.

**FIGURE 6:**
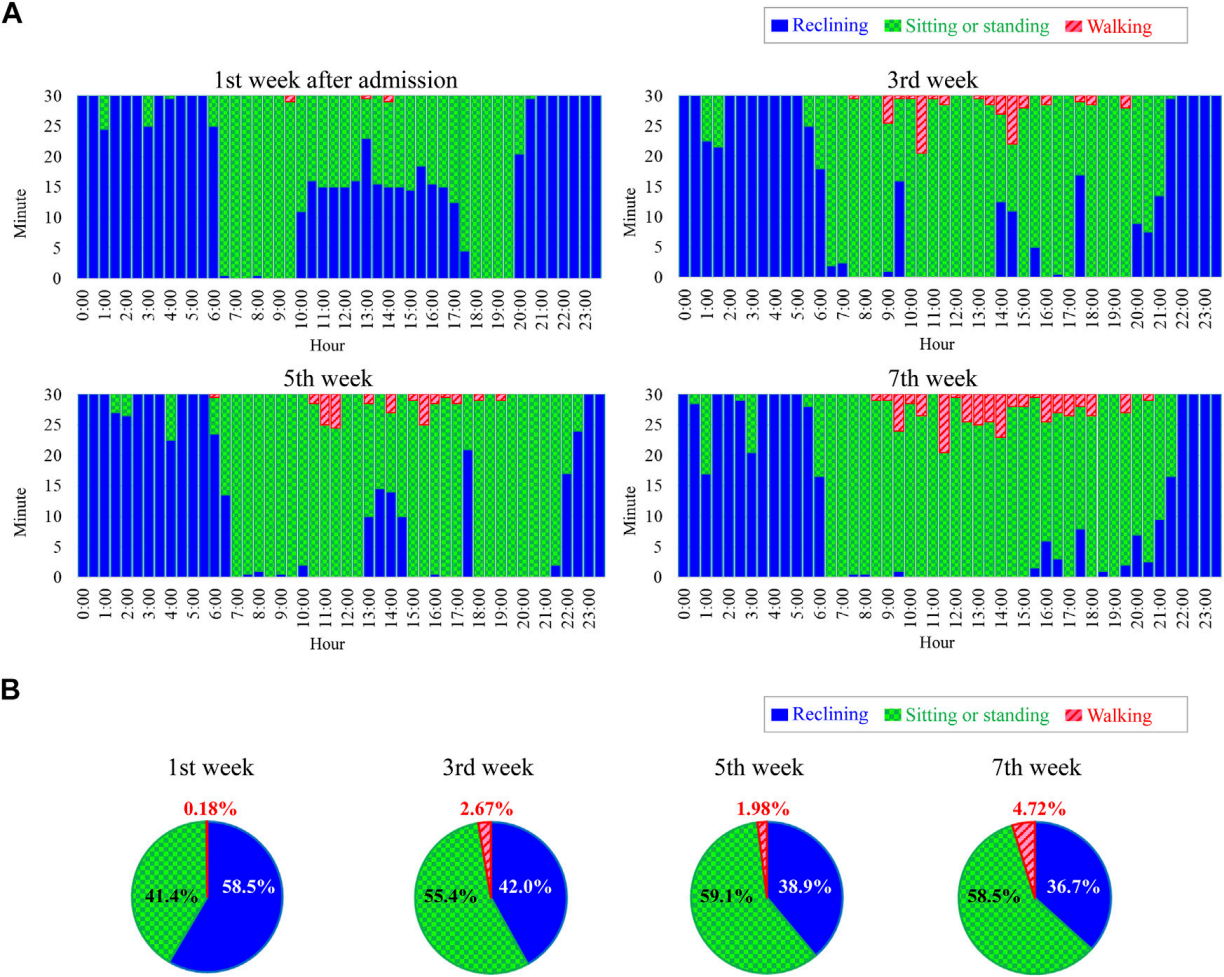
Example of postures of a stroke patient during 24 h. **(A)** Ensemble averaged postures from 1st week to 7th week after admission. **(B)** Ratios of total periods of each posture during 24 h.

Like the postures of all patients during 24 h, time series of individual stroke patients can also be visualized with the ensemble averaging method. Ensemble-averaged postures from the 1st week to the 7th week after admission are shown in Figure 6. In Figure 6A, reclining periods in daytime which were particularly long in the 1st week had decreased by the 7th week. Moreover, periods of sitting or standing as well as walking tended to increase as the weeks passed. This observation is in accord with the change in the ratios of the total periods for each posture during 24 h calculated using (4) (Figure 6B). These results suggest that visualization of ensemble-averaged posture allows us to see in detail changes in the activity of individual stroke rehabilitation patients.

## 5 Discussion

Here, we discuss the potential advantages and limitations of the ensemble-averaging method.

### 5.1 Potential advantages of ensemble averaging

#### 5.1.1 Visualization of imputed posture

The postures shown in Figure 5 suggest that the ratios of each posture vary depending on the hour of the day. Such information can reveal typical daily activity patterns of patients and may give clues for establishing interventions to increase the daily amount of activity. Furthermore, such visualized posture information may be used as feedback for patients to help them in activities considered to be effective in improving muscle strength and exercise tolerance (James et al., 2012; Mok et al., 2019). The results in Figure 6 suggest that the proposed method successfully visualized the 24-h activity of an individual patient. This ability may lead to personalized medicine. The scheduled tasks and training menus of inpatients differ from day to day (Hutcheon et al., 2010), and ensemble averaging may decrease the effect of such day-to-day variations in the measured data. Furthermore, the data in Figure 6A show waking at night (0:00–4:00) events, which suggests that ensemble-averaged postures can be used to identify sleep disorders.

#### 5.1.2 Application to small samples

As mentioned in Section 1, previous medical studies on data imputation have used the multiple imputation method or machine-learning approach. Compared with these methods, the proposed method would have advantages in experiments with small samples or a few variables. Furthermore, because the proposed method is not an alternative to them, its ensemble averaging could be used as a pre-processing for them. The effectiveness of combining ensemble averaging with the previous methods should be evaluated in a future study.

### 5.2 Limitations

#### 5.2.1 Cases in which proposed method is ineffective

There are two cases in which the proposed ensemble averaging might not be effective. First, lost data were not imputed so well when a large amount of data was lost during most of the measurement days. The result shown in Figure 3 suggests that data were not well imputed when the missing data rate was more than 50%. Second, our method will not work well when even small amounts of data are lost at the same time of day on all measurement days. Although Figure 4; Supplementary Figure S4 suggest that the proposed imputation method is effective to data loss that randomly occurs, the effect would be limited to such losses that repeatedly occur at the same time of day. Even in such cases, however, the results shown in Figure 4 suggest that the ratios for each posture will stay in the certain range independently of the period of data loss.

#### 5.2.2 Regularity of habits and physical condition of subject

The correctness of using ensemble averaging to generate posture information remains an issue. Although the proposed method is not always applicable, we think it is a good choice when the research target conforms to the following two cases. One case is when the daily activities and habits of the subjects are expected to be similar to the ones they showed during the measurement session. If irregular or rare events occur during the same measurement session, the ensemble-averaged postures will not represent the postures of typical activities. The activity patterns on weekdays are generally different from those on the weekend (Willetts et al., 2018; Matsuura et al., 2019), and the effect of the day of the week on the proposed imputation method should be further investigated in the future. However, this study was conducted in a convalescent rehabilitation ward in Japan, where rehabilitation services are provided 365 days a year, regardless of weekdays or weekends. Thus, its results show the fundamental performance of the proposed imputation method because the validation was conducted with the data containing simple activity pattern that was not influenced by the difference between weekday and weekend. The other case is when it is expected that the physical condition of the subject will not drastically change during the measurement session. For example, the recovery of some patients, especially those in the acute phase, progresses rapidly week by week, so their posture during 24 h will change depending on the recovery phase. Thus, the measurement periods for ensemble averaging should be limited to within a few days during which the physical condition of the patient is not expected to change significantly. In our experiment, we measured patients in a hospital at times when no irregular events were scheduled and performed the ensemble averaging on two measurement sessions conforming to the above two cases.

## 6 Conclusion

This study presented an ensemble averaging method that enables imputation of categorical variables during a long-term recording. Posture is used as the categorical variable. The method imputes lost data individually, requiring no other variables besides posture. It outputs a time series of postures over 24 h with less lost data by calculating ratios of postures at the same time of day during several measurement-session days. The proposed method reduced the missing data rate significantly in comparison with a conventional method. In addition, we visualized the 24-h behaviour of all inpatients and individual patients by using the imputed posture data. Furthermore, in the process to validate the proposed imputation method, we preliminarily estimated that 35 and 143 degrees of trunk angle are possible thresholds with which to classify postures for stroke inpatients. The classification method for postures and the proposed imputation method would be useful for a long-term activity monitoring using consumer wearable devices.

## Data Availability

The raw data supporting the conclusion of this article will be made available by the authors, without undue reservation.
