# Supplementary material for: Ensemble averaging for categorical variables: Validation study of imputing lost data in 24-h recorded postures of inpatients
Source: Front Physiol. 2023 Jan 26;14:1094946. doi: 10.3389/fphys.2023.1094946 (PMC9910696; doi:10.3389/fphys.2023.1094946)
Supplement: Supplementary file 1 [file Presentation1.pdf]

Supplemental method 1 and result 1

The measurement session period, which is necessary for ensemble averaging in order to impute data sufficiently, was investigated.

1 Supplemental Method 1

Six healthy subjects (all males; mean age, 30 ±4 years) participated in this study. They agreed to wear the measuring devices during the entire four-day measurement session after checking the size and fit of the wearable device.

2 Supplemental Result 1

The imputation of the missing data by ensemble averaging was evaluated with the six healthy participants. We tested different periods of ensemble averaging. The results are shown in Supplemental figure 1. The median of the missing data rate was 6.1% of the 24 hours (88 minutes) without any imputation method. It decreased to 0.14 % (2.0 minutes), 0.0 % (0 minutes), and 0.0 % (0 minutes) periods of data loss when ensemble averaging was applied to two to four days of measurement data. Significance with a paired t-test was confirmed between the median value on day 1 and those on more than two days.

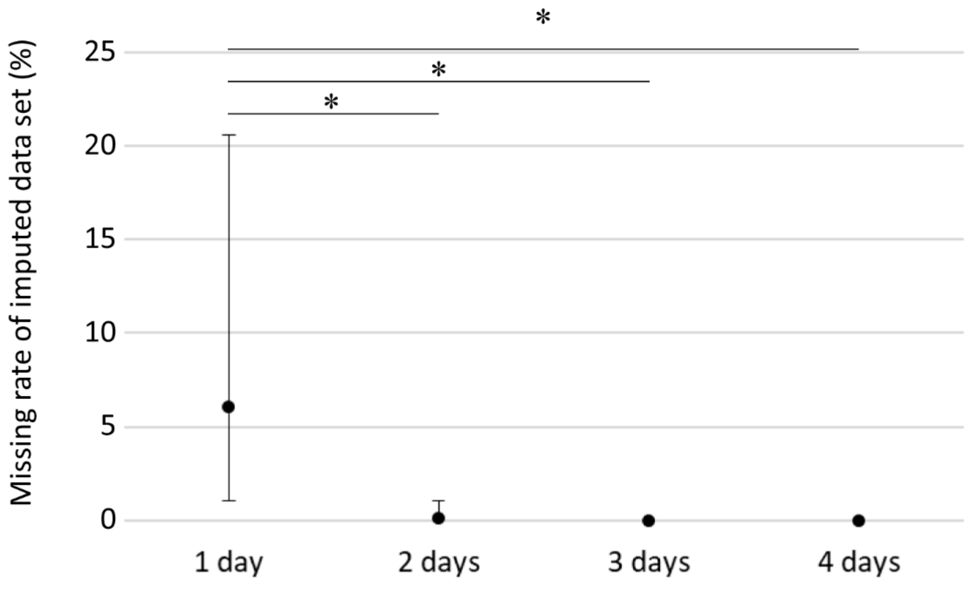

Supplemental Figure 1. Missing data rates when ensemble averaging is applied to two to four days of measurement data. The medians are plotted as points. Whiskers show the maximum and minimum values. Paired t-tests were conducted to test significance: *$p < 0.05$.

Supplemental method 2 and result 2

The possible thresholds of the trunk angles of stroke inpatients obtained during a two-day measurement were experimentally estimated.

3 Supplemental Method 2

The dataset containing acceleration data of 172 measurements successfully recorded for over 23 hours per day during a two-day measurement session.

4 Supplemental Result 2

Supplemental Figure 2 shows an example of the distribution of chest angles of a patient during a two-day measurement. The horizontal axis represents trunk angle, and the vertical axis represents frequency on a logarithmic scale. The vertical axis is in units of minute; thus, the value is the total time for each angle in the experiment. The box at the bottom shows postures at corresponding angles. When the trunk of the patient is upright, the angle is around 90 degrees in the calculation of equation (1). When the patient is prone, the angle is around 0 degrees, or 180 degrees in the supine position. There are two valleys on the histogram, and the possible thresholds to classify postures should lie in the valleys. Thus, we manually set thresholds at the middles of the valleys. Supplemental Table 1 lists the averages and 95% confidence intervals of the thresholds. The number of samples is 81 for the threshold between prone and upright and 172 for that between upright and prone. The numbers of samples were different because of a participant who did not take the prone position during the experiment. The results in the table suggest 35 degrees as the possible threshold between the prone and upright postures and 143 degrees as the one between upright and supine postures for stroke inpatients.

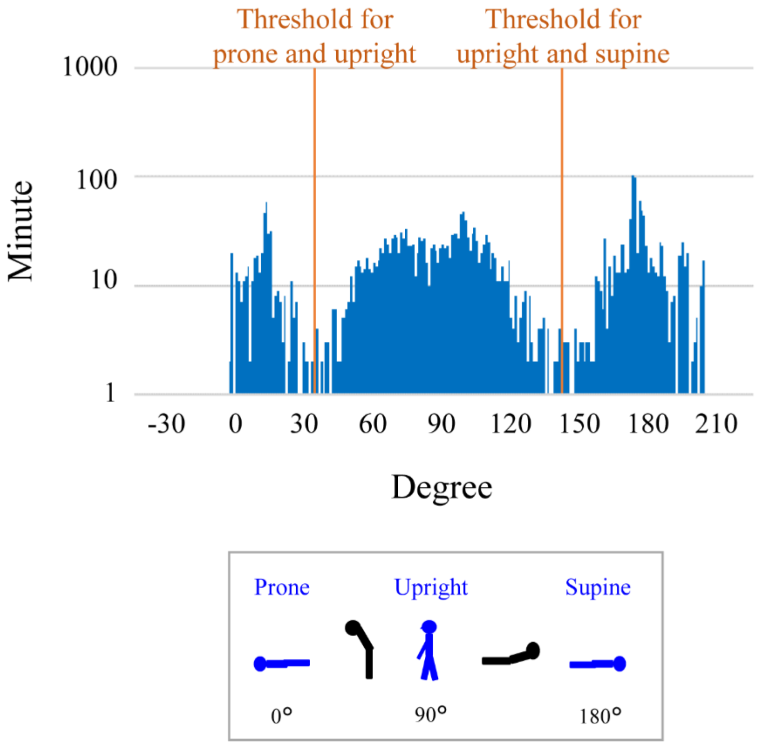

Supplemental Figure 2.

Example of distribution of chest angles of a patient during a two-day measurement. The horizontal axis represents trunk angle, and the vertical axis represents frequency on a logarithmic scale. The vertical axis is in units of minute. The box at the bottom shows postures at corresponding angles. The two lines in the local valleys are examples of thresholds determined manually.

Tables

Supplemental Table 1: Values of the threshold to classify postures.

**Threshold between:**	**Average and 95% confidence interval (degree)**	**Number of samples**
Prone and upright	35.4 (33.7 – 37.1)	81
Upright and supine	142.7 (141.5 – 143.8)	172

Supplemental method 3 and result 3

The effect of the duration of the data loss on the proposed imputation method was evaluated.

5 Supplemental Method 3

The validation was conducted with a two-step procedure. First, to confirm the statistics of the data loss in the entire data set obtained in this study (n=306), their duration and frequency were investigated. Second, the effect of the duration of the data loss on the proposed imputation method was validated, varying the duration of data loss. In the second step, the test dataset containing acceleration data of 172 measurements successfully recorded for over 23 hours per day during a two-day measurement session was used in the same way as described in Section 4.2.

6 Supplemental Result 3

Supplemental Figure 3 shows the number of occurrences of data loss during 24 hours per subject. In this study, most of the losses in the data recording lasted for 10 minutes or less. Such losses occurred 54 times on average during 24 hours for each participant and were the most frequent (98.4%) among the entire loss. This result suggests that the effect of data losses with durations of more than 10 minutes can be ignored when a data imputation method is validated with data of inpatients recorded with wearable devises.

On the basis of the results shown in Supplemental Figure 3, we added artificial data losses with different durations to the test dataset. Supplemental Figure 4 shows total periods and ratios of each posture taken during 24 hours with imputation. Durations of 1 and 10 min. were used as possible cases, and those of 50 and 100 min. were used to check to the performance of the proposed imputation method. The imputation performance showed no observable differences over the total period of each posture (Supplemental Figure 4A-C); neither were there any differences in the ratios of each posture over the total activity periods (Supplemental Figure 4D-F). These results suggest the effectiveness of the proposed imputation method to data losses that randomly occur.

Supplemental Figure 5 shows a specific example of patient data with artificially added losses with durations of 1 and 10 minutes. Supplemental Figure 5A shows an example without artificial data loss. There was no data loss due to the proposed imputation method. To the example, artificial data losses were added at a missing rate of 12.4%, which was the 75th percentile of the missing data rate of the original dataset shown in Figure 4. Supplemental Figure 5B and 5C show the results for artificial data losses with durations of 1 and 10 minutes, respectively. After the proposed imputation method was performed, the ratio of the period of total activity data and remaining loss during 24 hours was the same as 98: 2 in 5B and 5C even though the data-loss durations were different.

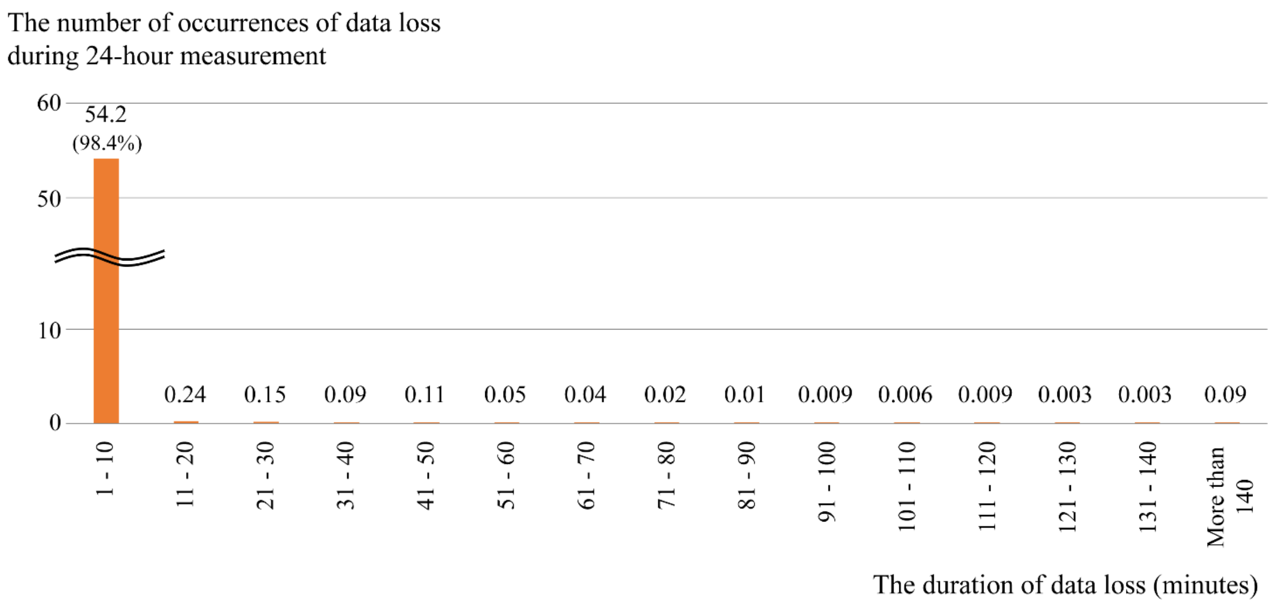

Supplemental Figure 3.

The number of occurrences of data loss during 24 hours per subject. The dataset containing all of the acceleration data collected in the 306 measurements was used.

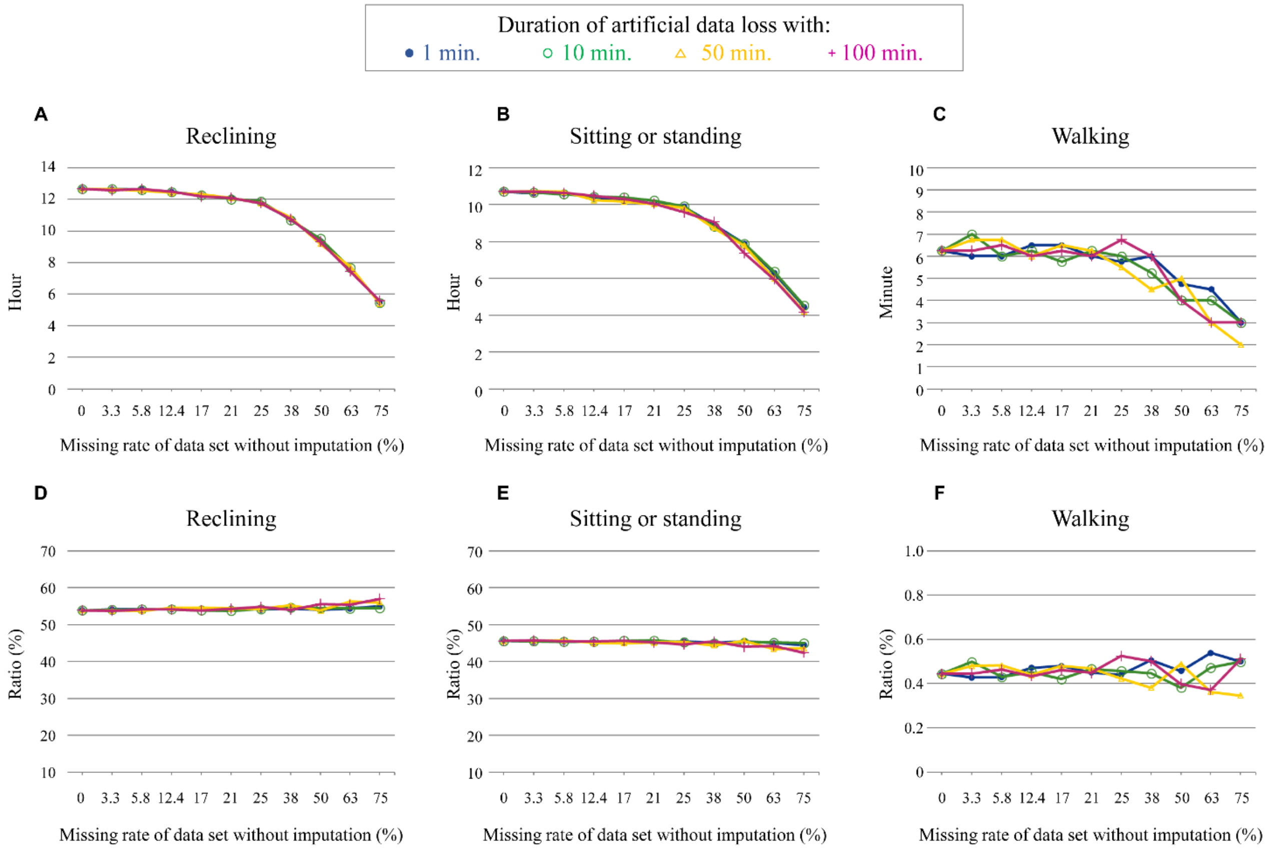

Supplemental Figure 4.

Total periods and ratios of each posture taken during 24 hours with imputation performed. (A) Total period of reclining, (B) sitting or standing, (C) walking posture during 24 hours. (D) Ratios of period of reclining, (E) sitting or standing, (F) walking posture over the total activity period.

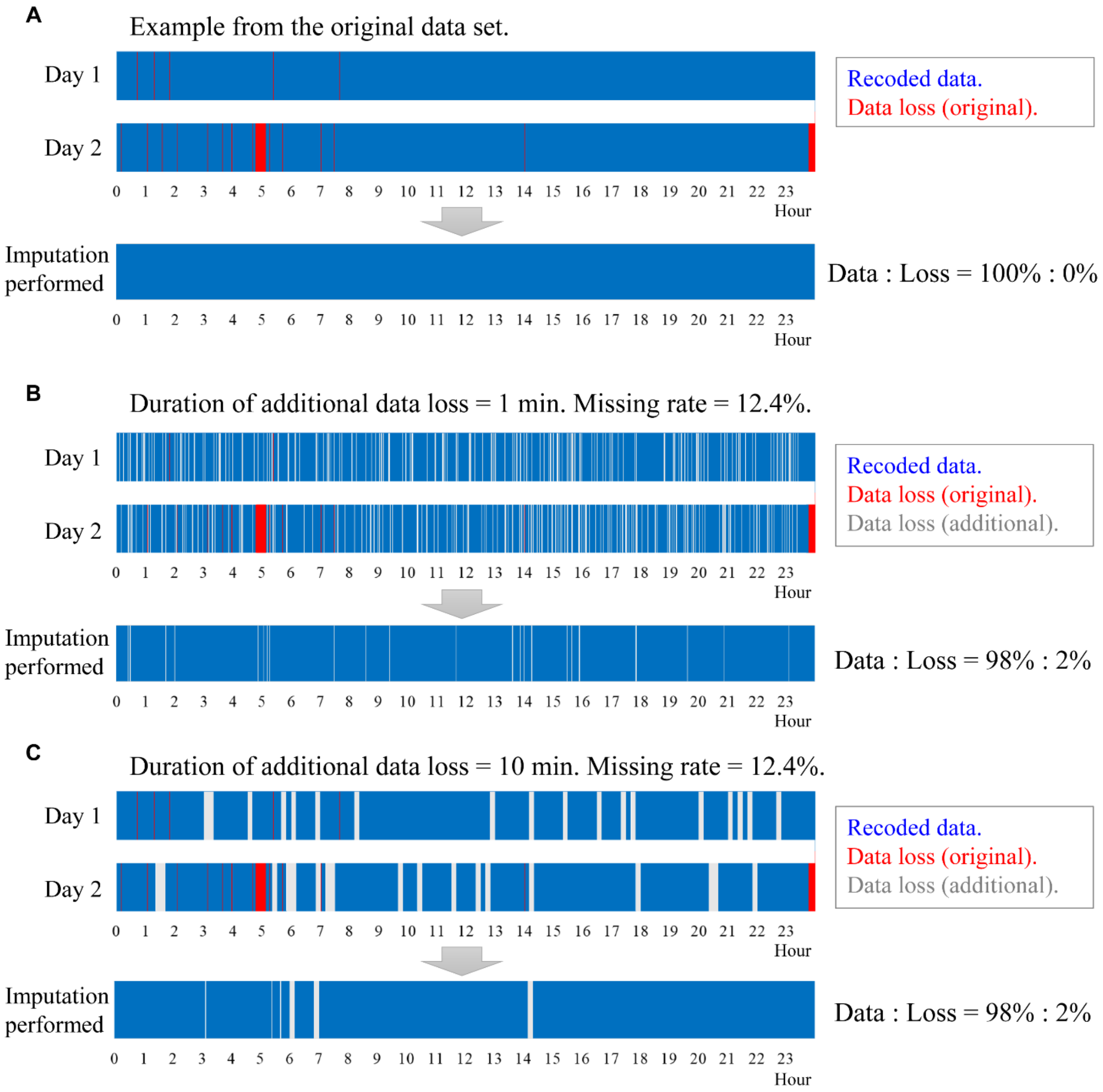

Supplemental Figure 5.

Specific example of patient data with loss artificially added with durations of 1 and 10 minutes. (A) Example of patient data without artificial data loss. There was no data loss after the proposed imputation method was performed. (B) Result with artificial data loss of 1 minute duration. (C) Result with artificial data loss of 10 minute duration. Missing rate was 12.4% in both (B) and (C).

Supplemental discussion

Here, we discuss the estimation of the body-angle threshold that was used to classify posture.

Posture Classification

Two local valleys in the histogram of trunk angle were found around 35 degrees and 143 degrees (Supplemental Figure 2); the former can be used as the threshold to classify the prone and standing positions, and the latter can be used as the one between the standing and supine positions.

There are two reasons for the appearance of these valleys. One is that there were specific postures typically taken by hospital inpatients. In Supplemental Table 1, three clusters of angle appear at the upright, prone and supine positions. The angle values are distributed in each cluster because of individual differences in body shape and flexion or extension of the spine. The second reason has to do with balancing the weight of the upper body. It is hard to maintain balance when the center of mass drifts outside the base of support. Because such a drift leads to a fall (Horak et al., 1997), the occurrence of angles in unbalanced postures would not be frequent. In other words, the valleys may have been a consequence of the patient avoiding such unbalanced postures.
